# Reminiscences of the Insane

**Published:** 1886-12-18

**Authors:** 


(*To be continued.*)

### Reminiscences of the Insane.

By a Mental Physician.

**Lunatic Critique on a Lunatic.**

The conversations between patients themselves in regard to their delusions are often singular in the extreme. One evening, in the library of the asylum, we overheard a patient, who was reading W. Wilberforce's Life by his sons, observe, "I used to know William very well." Another patient replied, with a sarcastic manner, "Who, John?" "William Wilberforce" was firmly replied. The critic observed, smiling, "Oh, indeed." Upon this a third patient joined in with, "Hold your tongue, with you; speak the truth when you do open your mouth." The critic put in, "When did you know him?" The patient replied, unabashed, "Oh, during his parliamentary career. It would be about 1800, or thereabouts."

**Dreaming v. Insanity.**

This patient frequently migrated into centuries long passed, and undoubtedly felt that he was in one of the scenes enacted. The analogy between states of insanity and dreaming is illustrated in a remarkable manner in such patients. Now they live in the age of Elizabeth and are familiar with the great men of her day. At another time they are living in the reign of Charles the First, take an active part in the Civil War, and have witnessed the execution of the royal prisoner at Whitehall. As in dreams, they see no inconsistency between all this and the objects and people and places around them. The sense of anachronism is altogether wanting; indeed, time is abolished. Space also, for such patients mix up their geography, while describing their travels, with as charming a disregard of distance as the historian of King Arthur and the travels of his knights in quest of the Holy Grail. During the same evening our patient, who had strong suspicions of foul play and subterraneous influences, remarked, "I want an investigation into these flying gentlemen who are going backwards and forwards about me." "What do you mean?" said the critic. Patient: "Well, you know, there are several Englands, and several Cumberlands, and so with people, several are as much alike as a likeness could be."

**A Contest of Rival Delusions.**

It is not at all an uncommon thing for two patients with identical delusions to quarrel with one another in consequence. This is never more striking than when patients labour under the delusion of being themselves the Persons of the Trinity, and each demands from the other the worship which this very remarkable delusion demands. Almost every asylum has its kings and queens who are offended with the assumption by others of the royalty to which they themselves aspire and claim. Lord Chancellors figure also in this motley assemblage; and it is a fact that when, on one occasion, a real Lord Chancellor presented himself at the gates of a public asylum, and, on being asked his name, replied "The Lord Chancellor," the porter, supposing him to be labouring under a delusion, replied, "We have too many of them already."

**Unconscious Inconsistency of Patients.**

We have spoken of the inconsistencies and incongruities of the thoughts and acts of patients, and the analogy between these and the scenes of our dream-life. The following illustration of insane inconsistency ought not to be omitted. It occurred in a patient who believed himself to belong to the noblest families in the land, and that, like the late Mrs. Girling, he should never die. One day we heard him assert, in reference to his beard, which he wore before beards came into fashion, "each hair is possessed of a distinct intelligence, which enables it to arrange itself to the best advantage without any care on my part." Yet, with the inconsistency to which we refer, he was daily engaged in combing and brushing it. Up to the last moment of his life this patient protested that he would not die.

